# Machine learning identifies cytokine signatures of disease severity and autoantibody profiles in systemic lupus erythematosus – a pilot study

**DOI:** 10.1038/s41598-024-79978-9

**Published:** 2024-11-20

**Authors:** Sarit Sekhar Pattanaik, Bidyut Kumar Das, Rina Tripathy, Birendra Kumar Prusty, Manoj Kumar Parida, Saumya Ranjan Tripathy, Aditya Kumar Panda, Balachandran Ravindran, Ratnadeep Mukherjee

**Affiliations:** 1 Department of Clinical Immunology and Rheumatology, SCB Medical College, Cuttack, Odisha India; 2 Department of Biochemistry, SVP PG Institute of Paediatrics, Cuttack, Odisha India; 3 Institute of Life Sciences, Bhubaneswar, Odisha India; 4 Department of Bioscience & Bioinformatics, Berhampur University, Berhampur, Odisha India; 5 Section for Immunology, Division of Infection Control, Norwegian Institute of Public Health, Oslo, Norway

**Keywords:** Cytokines, Systemic lupus erythematosus, Machine learning

## Abstract

Disrupted cytokine networks and autoantibodies play an important role in the pathogenesis of systemic lupus erythematosus. However, conflicting reports and non-reproducibility have hindered progress regarding the translational potential of cytokines in SLE. This study attempts to address the existing knowledge gap using multiplex cytokine assay and machine learning. 67 SLE patients fulfilling SLICC criteria were recruited after informed consent, and circulating cytokines were measured by multiplex cytokine assay kit. We observed a positive association between actual disease activity scores (SLEDAI) and predicted scores from a partial least squares regression (PLSR) analysis of multivariate cytokine response data, with MIP-1α having a strong contribution towards disease activity. Our analysis also highlights increased IL-12 as a potential biomarker in nephritis and elevated MIP-1α as a signature of NPSLE. Using a k-Modes clustering algorithm to stratify patients based on patterns of co-occurrence of circulating autoantibodies, we identified 4 distinct clusters of patients. All 4 clusters had patients with nephritis, but patients in cluster 3 with nephritis were characterised by low levels of housekeeping and homeostatic cytokines and the presence of anti-Ro antibodies, which is a novel observation. Thus, we demonstrate that cytokines can be a surrogate to predict disease activity and organ involvement in SLE. Moreover, we show the utility of unsupervised machine learning algorithms using specific autoantibody signatures to predict renal involvement in SLE.

**Supplementary Information:**

The online version contains supplementary material available at 10.1038/s41598-024-79978-9.

## Introduction

Systemic lupus erythematosus (SLE) is a prototypic, multisystemic autoimmune disease, predominantly affecting females in the reproductive age group^1^. Our understanding of the disease has changed over the years, from being a disease of unknown aetiology to significant improvement in dissecting pathways driving disease pathogenesis resulting in new insights and therapeutic advances^2^. Despite a better understanding of pathogenesis and the availability of newer targeted therapies, SLE continues to be one of the leading causes of death in young females and is associated with considerable morbidity and poor quality of life^3^.

Genetic susceptibility, environmental triggers leading to immune dysregulation, autoantibody formation, and cytokine network disruption are key contributors to disease initiation and perpetuation^4^. The heterogeneity of clinical phenotypes in SLE is probably a reflection of different underlying immune pathways which are contributed by cytokines, immune cells, and autoantibodies. Cytokines, notably IFN-α, IL-6, IL-17, TNF- α, and their downstream signalling play an important role in the pathogenesis of the disease^2^. Understanding the precise role of cytokines in perpetuating the disease has led to novel therapeutic targets in SLE like Belimumab and Anifrolumab^5,6^. However, since there are multiple cytokines with pleiotropic effects, there are conflicting reports about their association with various clinical phenotypes^7–9^.

Several cytokines and chemokines are elevated in SLE due to ongoing immune activation and may contribute to active disease. IFN-α is an important regulator in the pathogenesis, contributing to disease activity^10^. Furthermore, levels of several other cytokines and chemokines like CXCL-10, PTX-3, BAFF, APRIL, IL-17, TNF-α, MIP1α, MIP1β, IL-8, MCP-1, IL-6, IL-8, IL-18, IFN-γ, and IL-10 are also elevated in patients with active SLE^11^.

SLEDAI, a combined score of clinical and serological parameters is a validated tool for assessing disease activity^12^. It requires trained manpower for assessment and in countries lacking trained rheumatologists, there may be error in scoring^13^. Additionally, serological markers like dsDNA and complements may remain persistently deranged in certain patients without clinical manifestations. That is the basis of categorising a group as clinically quiescent but serologically active^14^. A newer approach using cytokines can possibly overcome these issues which are still pertinent to resource limited setup country like India.

Nephritis, seen in 50–60% of Indian SLE patients, is a leading cause of morbidity and mortality^15–17^. Besides immune complex-mediated injury, infiltrating immune cells and cytokines also contribute to pathogenesis. IL-12, a key pro inflammatory Th1 Cytokine has been associated with disease activity and chronic changes on renal biopsy in patients with lupus nephritis^18–20^. Besides, other cytokines like IL-4, IL-2, IL-10, IFN-γ, and TNF-α have been shown to be elevated in patients with nephritis^11^.

Neuropsychiatric lupus (NPSLE) is another severe and heterogeneous clinical entity^21^. Differences in case definition and lack of good biomarkers have always posed a challenge in its management. IFN-α is believed to be the leading effector in the pathogenesis of NPSLE^22^. It has been shown to result in aberrant synaptic pruning leading to psychosis. IL-6 and IL-18 in CSF and serum have been associated with psychosis and seizures respectively^23,24^.

Autoantibodies play an important role in disease pathogenesis. Clustering of autoantibodies with various clinical phenotypes, like anti ds-DNA with nephritis and Sm/RNP with serositis have been reported^25–27^. However, there are conflicting reports regarding association with autoantibodies, cytokines and disease activity. A Colombian study showed a positive association between autoantibody clusters, cytokines, and disease activity^28^, a finding not corroborated in another Swedish SLE cohort^29^.

SLE is a multi-dimensional disease, associated with many non-linear variables. This pilot study attempts to combine clinical profiles, cytokines, and autoantibodies using a machine-learning approach to understand their complex relationship and identify potential biomarkers for severe clinical manifestations like nephritis and NPSLE.

## Results

### Study design and patient demographics

**Fig. 1:**
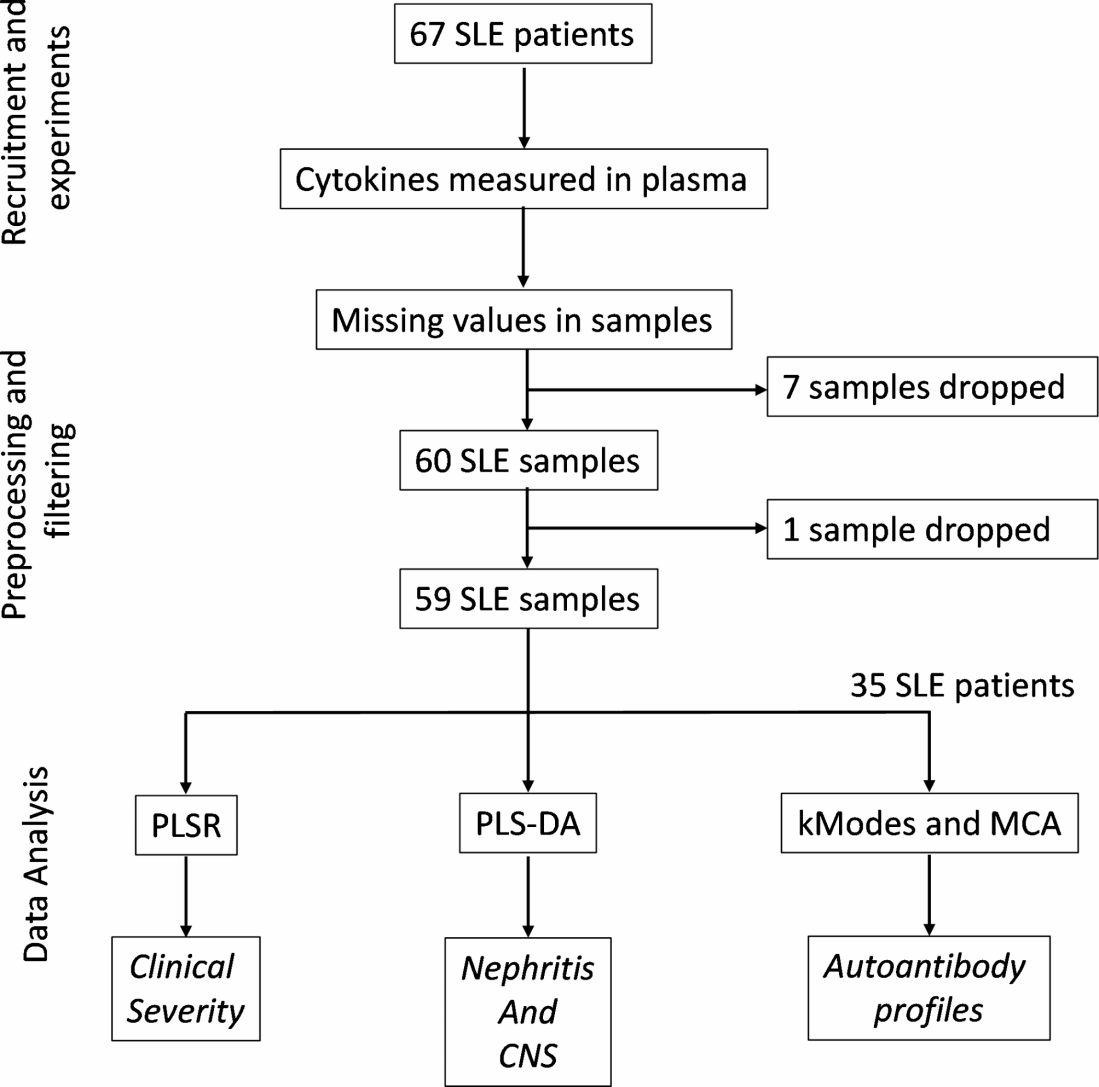
Flow chart depicting the experimental and analytical design of the study.

An overview of the experimental and analytical pipeline is shown in Fig. 1. Initially, 67 patients were recruited for the study (Table 1). The Female: Male ratio in our cohort was 10:1. Nephritis was the most common major organ involved seen in 61% of patients, followed by Neurological and Hematological involvement seen in 15% of patients. Most patients in our cohort had active disease as highlighted by a mean SLEDAI of 10.6 and significant damage with a mean SLICC ACR DI of 2 (Table 1). 7 samples were dropped because of a lack of signal from more than 10% of the measured cytokines. For detecting potential outliers, we created a correlation matrix of cytokines across all samples, which resulted in two clusters of cytokines that were strongly correlated (Supplementary Fig. S1). Pairwise scatterplots of one cluster of cytokines revealed abnormally high values for all cytokines in one patient (Supplementary Fig. S2). This individual was designated as an outlier and was dropped from further analysis. Our final analysis consisted of 59 SLE patients.

**Table 1:** Demographic and clinical characteristics of enrolled patients.

Parameter	SLE Patients (*n* = 67)
Gender (Male/Female)	6/61
Age (Mean ± SD)	28·4 ± 8·6
Disease duration (Mean year ± SD)	1·7 ± 2
SLEDAI (Mean ± SD)	10·6 ± 6·3
SLICC ACR DI (Mean ± SD)	2 ± 1·1
SLICC 2012 criteria	
Acute Cutaneous	38(57)
Chronic Cutaneous	4(6)
Oral/Nasal ulcer	43(64)
Non-Scarring Alopecia	39(58)
Arthritis	27(40)
Serositis	9(13)
Renal	41(61)
Neurologic	11(16)
Haemolytic Anaemia	10(15)
Leukopenia	1(2)
Thrombocytopenia	1(2)
Cardiovascular	4(6)
Respiratory	5(7)
Gastrointestinal	5(7)

Note: Data are given in number (%) unless otherwise specified, SD: Standard deviation.

Cytokines exist as interconnected networks, where groups of cytokines having similar functions are often found to co-occur. With this in mind, we wanted to check cytokine co-occurrence networks in both healthy controls and patients with SLE. As can be seen in Supplementary Fig. S3A, we observed strong correlations between the measured cytokines in healthy controls. Moreover, the cytokines also segregated into five natural clusters based on their co-occurrence. However, a similar analysis of circulating cytokines in SLE patients revealed a complete lack of any structure in their co-expression network, with almost every cytokine forming a separate cluster of its own (Supplementary Fig. S3B). This observation strongly suggest that SLE leads to a global disruption of cytokine networks.

### Cytokine markers can predict SLEDAI scores in SLE patients

**Fig. 2:**
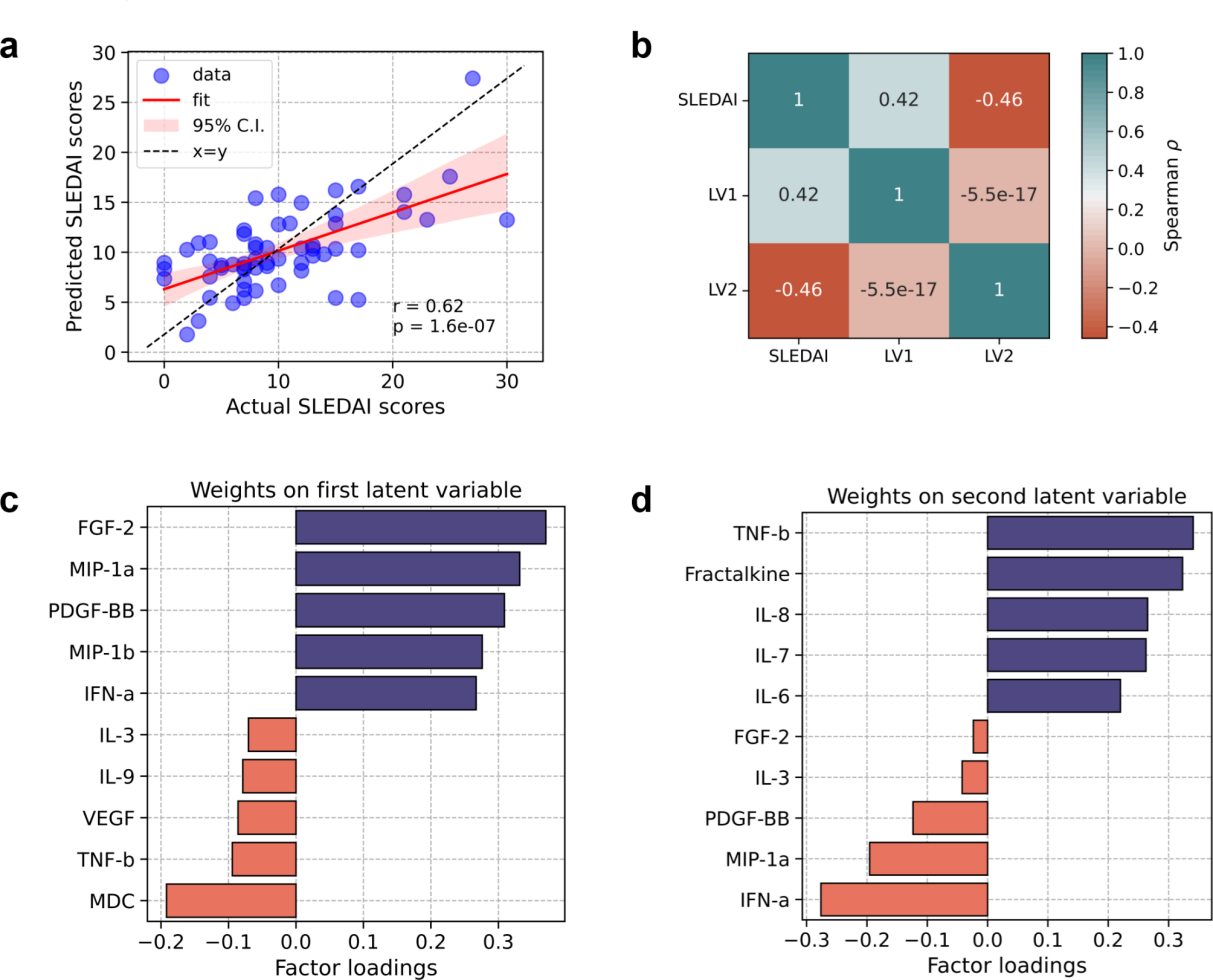
Combined cytokine response in SLE is predictive of disease severity. **(A)** Correlation between observed vs. predicted SLEDAI scores in patients with SLE. The SLEDAI scores were predicted from a PLSR model with cytokines. **(B)** Correlation of SLEDAI scores with the first two components of the PLSR model (LV1 and LV2). **(C)** Cytokine contributions towards the first PLSR component (LV1: Latent variable 1). **(D)** Cytokine contributions towards the second PLSR component (LV2: Latent variable 2).

Our first objective was to test if cytokine profiles in SLE can predict disease severity. To this end, we performed partial least squares regression (PLSR) analysis with the cytokine levels as predictors for disease activity score (SLEDAI). We observed a moderate positive correlation between predicted and observed SLEDAI scores (Fig. 2a, spearman ρ = 0·62, p-value = 1·6e-07). To identify which cytokines were the best predictors, we first performed a correlation analysis of the latent variable scores for the first two components from the PLSR model with the observed SLEDAI scores, which revealed a positive correlation with the first PLSR component (LV1) and a negative correlation with the second PLSR component (LV2) (Fig. 2b). Further analysis of the contribution of individual cytokines toward LV1 and LV2 showed that FGF-2 was the strongest positive contributor to LV1, with MIP-1α being the second most important (Fig. 2c). PDGF-BB and IFN-α were also found to be positively associated with disease severity (higher positive contribution towards LV1). On the other hand, cytokines like MDC and TNF-β were the top two negative contributors. The roles of TNF-β and MIP-1α were reversed when we assessed the factor loadings for LV2 (Fig. 2d), suggesting their reciprocal effects on SLE-associated disease severity.

### Multivariate analysis of cytokine expression can differentiate SLE patients with or without nephritis or CNS involvement 

**Fig. 3:**
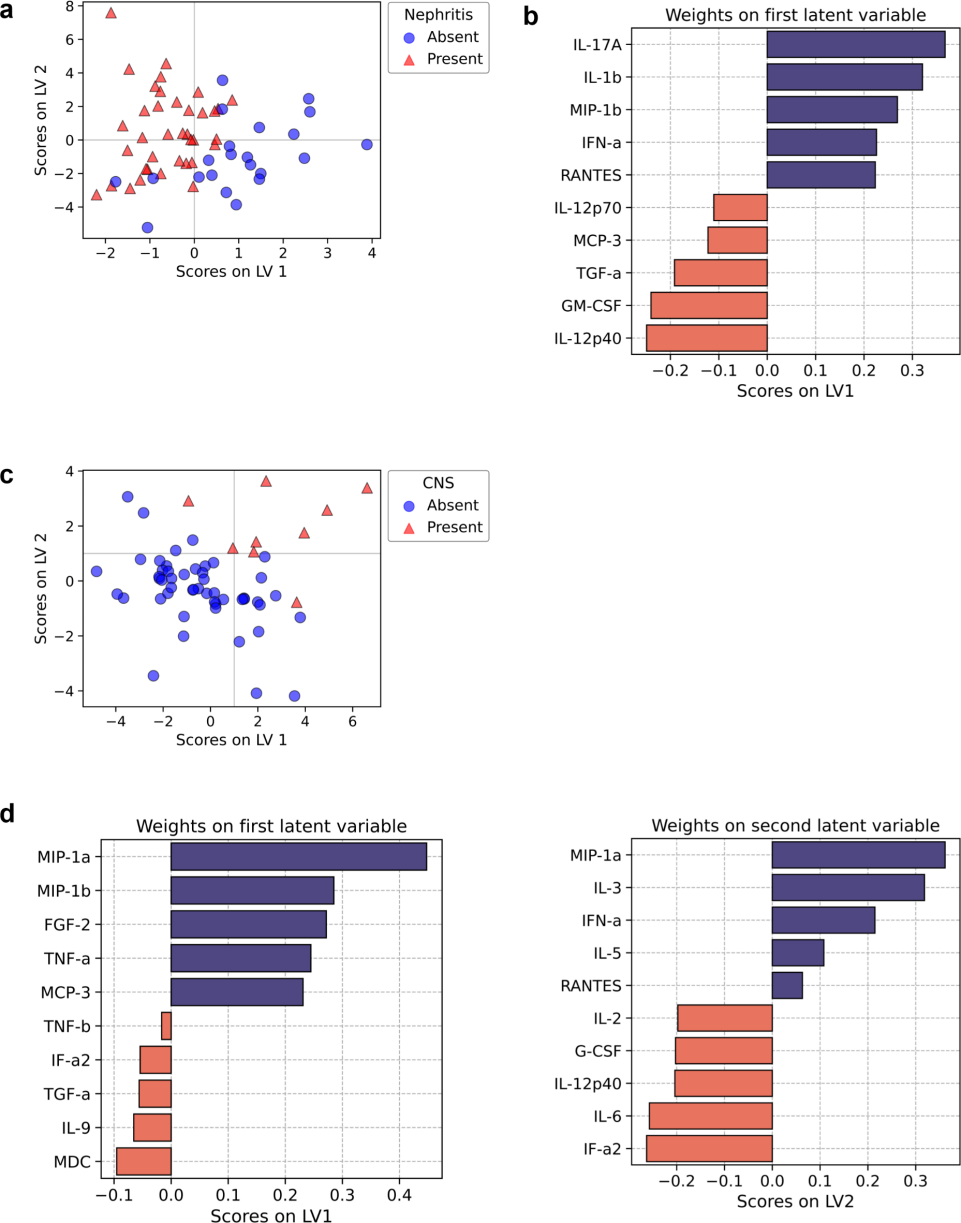
Cytokine markers of nephritis and CNS involvement in SLE. **(A)** Partial Least Squares Discriminant Analysis (PLS-DA) of cytokine response and subsequent projection into latent variable space discriminates patients with and without nephritis in SLE. **(B)** Top five positive and negative cytokine contributors towards the first latent variable that discriminates patients with or without nephritis. **(C)** Partial Least Squares Discriminant Analysis (PLS-DA) of cytokine response and subsequent projection into latent variable space discriminates patients with and without CNS involvement in SLE. **(D)** Top five positive and negative cytokine contributors towards the first and second latent variables discriminating patients with or without CNS involvement.

Nephritis and neurological manifestations are two of the most severe forms of SLE. Therefore, our next goal was to test whether cytokines could stratify these patients. We first performed a PLS-DA on patients with or without lupus nephritis. Projection of the individuals on the 2D space of the first two latent variables shows a clear separation of patients with or without kidney involvement along the first latent variable (Fig. 3a), where patients without nephritis were seen to have higher LV1 scores as compared to patients with nephritis. Examination of contribution of cytokines toward LV1 revealed high positive scores for IL-17 A and IL-1β, whereas IL-12p40 and GM-CSF were the two most important negative contributors (Fig. 3b). This suggests that high IL-12p40:IL-17 A ratio is a signature of SLE-associated nephritis. A similar analysis for patients with neurological manifestations in the form of psychosis and seizure scored high on both LV1 and LV2 (Fig. 3c). We, therefore, inspected the factor loadings for both LV1 and LV2. The plot in Fig. 3d shows that MIP-1α is a strong contributor to higher LV1 and LV2 scores, suggesting its potential role as a novel marker for SLE-associated neurological complications.

### A machine learning-based algorithm identifies distinct clusters of patients based on unique signatures of circulating autoantibodies

**Fig. 4:**
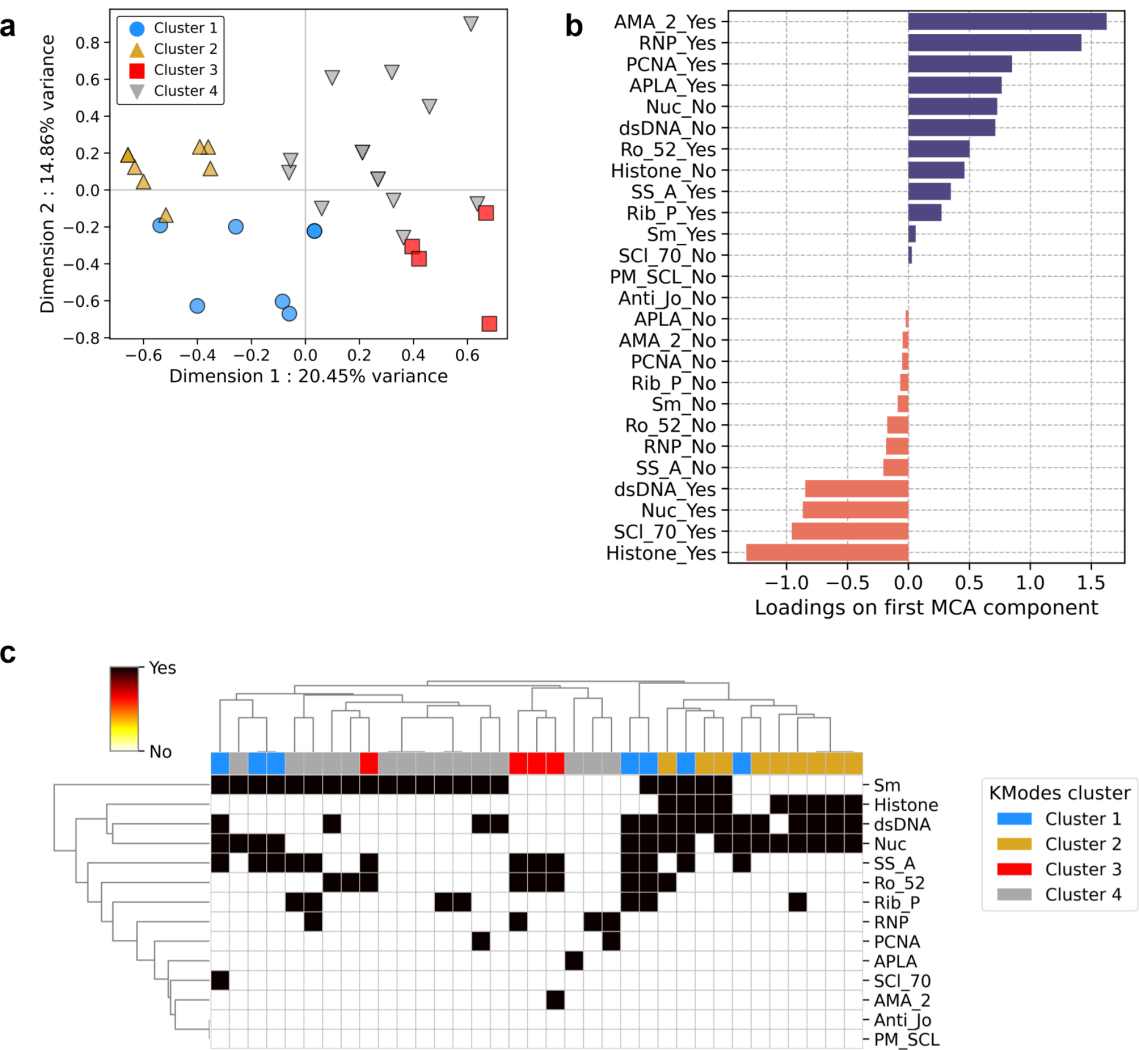
SLE patients have distinct combinations of autoantibody profiles having separate cytokine signatures. **(A)** KModes clustering algorithm identifies 4 distinct clusters of patients based on combined autoantibody profiles. The patients are then visualized on a 2D representation of the multidimensional data obtained by multiple correspondence analysis (MCA). **(B)** Factor loadings plot demonstrating the relative contributions of the individual autoantibodies to the separation observed in **(A)**. **(C)** Hierarchical clustering heatmap of patients stratified by their autoantibody profiles. Black demonstrates the presence of an autoantibody while white depicts its absence. The natural clusters agree well with the clusters found by the Kmodes algorithm, which is used to color-code the patients.

There is limited knowledge about the combinations of autoantibody profiles and their association with clinical phenotypes. We, therefore, investigated if patients in our study cohort can be stratified based on their autoantibody profiles. Since autoantibody profiles are categorical, traditional machine learning–based classifiers are not meaningful. For analyzing categorical autoantibody responses, we used the k Modes algorithm, which is a modification of the traditional k Means algorithm in that it uses the mode of the categorical attributes to define the cluster centers. The algorithm aims to minimize the total distance (or dissimilarity) between data points and their assigned cluster centers, where the distance is measured using a dissimilarity metric appropriate for categorical data. Using kModes clustering on combined autoantibody profiles, we were able to split the patients into 4 distinct clusters, which were chosen based on an elbow plot of reduction in the cost function of the model with increasing numbers of clusters (Supplementary Fig. S4). The clusters thus formed were visualized on a two-dimensional representation of the multivariate autoantibody dataset (Fig. 4a). The 2D representation was obtained by performing a multiple correspondence analysis (MCA), which is a dimensionality reduction method for categorical data. Our results show clear separations between the four clusters in MCA space, with clusters 2 and 3 being the farthest apart (Fig. 4a) on the first MCA axis, which accounts for the highest variance in the data (20·45%). We then investigated the factor loadings plot for the first MCA component that revealed a high negative contribution of the presence of anti-histone antibodies (Fig. 4b**)** To confirm our observations, we performed a hierarchical clustering of the patients according to their autoantibody profiles and color-coded them as per the clusters obtained by the kModes algorithm. As can be seen in Fig. 4c, hierarchical clustering recapitulates the clusters obtained from the kModes run, with clusters 2 and 3 showing the best separation with some overlap from clusters 1 and 4. Moreover, the clustering heatmap also revealed that patients belonging to cluster 3 are completely devoid of autoantibodies against histone, dsDNA, and nucleosome, while being positive for Ro/SSA autoantibodies. Contrarily, individuals clustered into group 2 had the exact opposite profile.

Taken together, our analysis suggests SLE patients can be classified according to their autoantibody profiles. We observed two clusters providing a clear dichotomy between the presence of either the triumvirate of anti-histone, dsDNA, and nucleosome autoantibodies or the presence of anti-Ro/SSA autoantibodies.

### Autoantibody profile in SLE is associated with different clinical manifestations and cytokine signatures

**Fig. 5:**
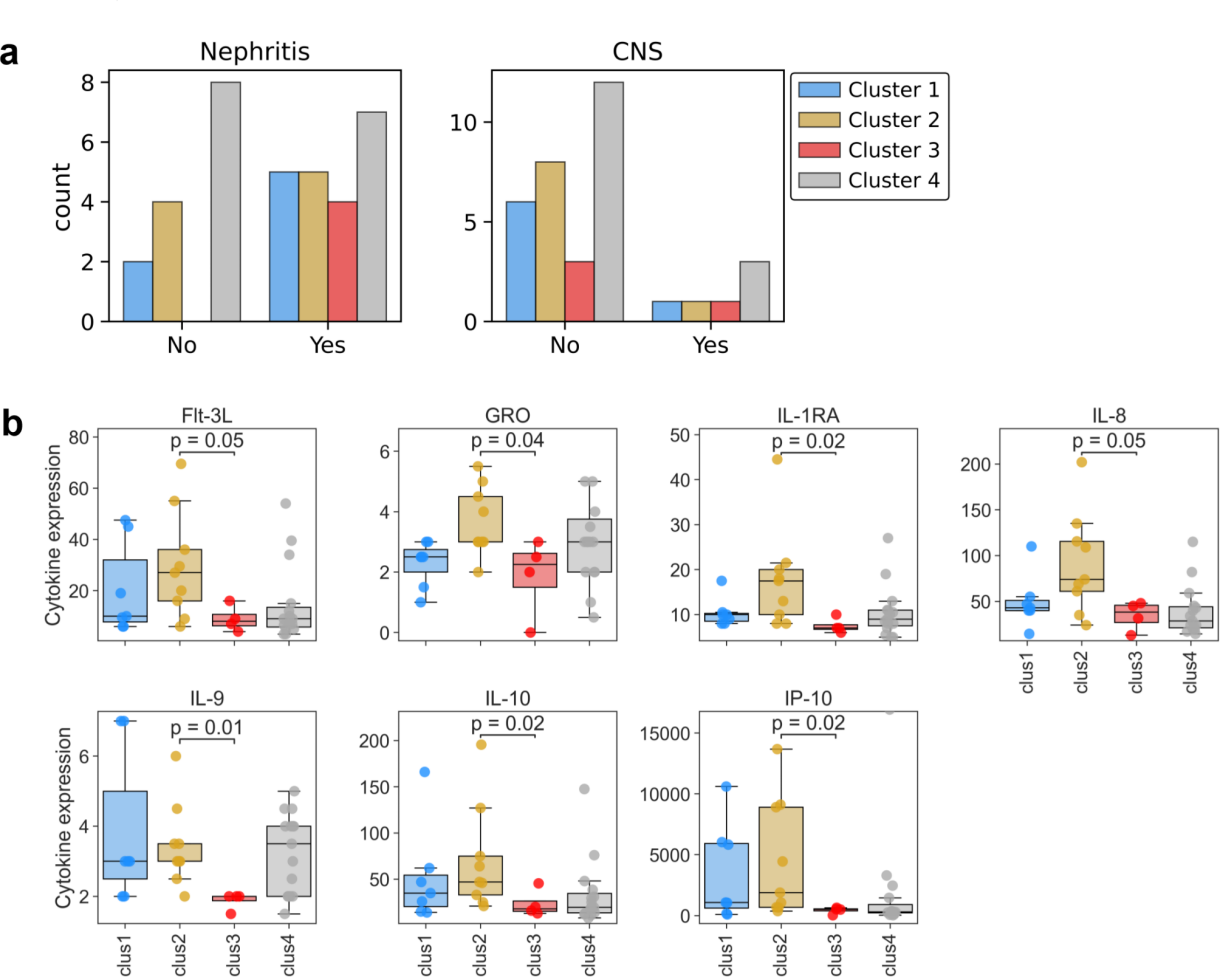
Autoantibody profiles in SLE are associated with organ involvement and distinct cytokine signatures. **(A)** Counts of patients presenting with or without lupus-associated nephritis or CNS involvement within the corresponding autoantibody clusters. **(B)** Cytokine profiles associated with autoantibody clusters. Comparison of cytokine levels between Clusters 2 and 3 was done by a Mann-Whitney U test, corrected for multiple tests by the Bonferroni method. Only significantly different cytokines are shown (*p* < 0.05).

Our next objective was to investigate whether the clusters obtained by unique autoantibody signatures have differences in clinical manifestations. An initial comparison of the mean SLEDAI scores of the clusters of patients as indicated below revealed no significant differences between the four clusters (Cluster 1 = 12, cluster 2 = 8.56, Cluster 3 = 10.75, Cluster 4 = 11.47). However, closer inspection of the distribution of lupus nephritis and NPSLE among the clusters revealed interesting differences. As can be seen in Fig. 5a, there were fewer patients with neurological involvement than those without neurological involvement irrespective of the autoantibodies observed. All antibody clusters had similar number of patients with nephritis. Interestingly, we found that all patients in cluster 3 had nephritis (Fig. 5a), and significantly lower levels of cytokines responsible for downregulating inflammation and maintaining cellular homeostasis (Fig. 5b) and anti-Ro/SSA antibodies (Fig. 4c). This appears to be a novel cluster that shows a possible association of anti-Ro antibodies with nephritis.

## Discussion

The current study of lupus patients from Eastern India, adapting a systems biology and machine learning approach for an integrated analysis of clinical phenotype, multiplex cytokine data, and autoantibody profiles generated some interesting observations. There is a global disruption of cytokines in SLE, with specific cytokines predicting disease activity and major organ involvement in the form of nephritis and NPSLE. Interestingly, all patients in a defined cluster with low levels of homeostatic and anti-inflammatory cytokines had concomitant presence of anti-Ro and nephritis. From the above observations, we conclude that autoantibody profiles in SLE are associated with unique cytokine signatures, which can help in predicting and stratifying severe manifestations: SLE nephritis could be unique in that context.

Disease activity in SLE contributes to morbidity and early mortality^30^. SLEDAI, a composite score of clinical and serological parameters is a widely validated and accepted metric for assessing disease activity^12^. Assessment of SLEDAI requires trained manpower and laboratory parameters which can be discordant to clinical disease activity^14^. The results of the current study using PLSR validate two groups of cytokines, high levels of FGF-2 and MIP-1α and low levels of TNF-β and MDC to be associated with disease activity. This provide an alternative in form of cytokine signatures which can be used in a resource limited setting, to calculate disease activity^13^. Studies have shown MIP-1α, TNF-β, MDC to be associated with disease activity. MIP-1α and MCP-1 are chemotactic for T cells and dendritic cells and have been proposed to contribute to the systemic inflammation in SLE. But the precise pathways through which these molecules contribute to disease activity in SLE remain to be elucidated^11,31^. SLEDAI cannot be substituted by an analyses of cytokines but it can provide an alternative method of analysis with more precision, unlike clinical manifestations which can be subjective in the hands of untrained physicians^13^. With AI looming in the horizon in health care sector, cytokine assessment to predict disease activity and severity could be a more robust and objective alternative.

Lupus nephritis is a management challenge with mortality of 25% at 5 years and 10–15% progression to ESRD despite the best treatment^32^. Early diagnosis and appropriate management are vital in preventing irreversible renal injury. Apart from immune complex-mediated damage, the role of infiltrating immune cells, and activation of non-immune resident cells producing cytokines and chemokines are being recognized as key players for disease progression^32,33^. Our results highlight the role of IL-12p40 in nephritis. Earlier studies have shown IL-12 in sera, urine and in renal tissue to be associated with lupus nephritis^18,19^. Results from Japanese cohort highlights the role of IL-12 in proliferative nephritis, showing patients in IL-12 group to have high chronicity compared to BAFF and IFN-α subgroups^20^.

NPSLE remains an enigma encompassing varied clinical manifestations with limited understanding of underlying pathogenesis and therapeutic options. Studies have shown cytokines like IL-6, IL-18 and IFN-α play a major role in the pathogenesis of NPSLE^22–24^. Using PLS-DA analysis our results demonstrate that MIP- 1α is significantly associated with NPSLE, similar to a Japanese cohort^34^. MIP-1α is a chemokine responsible for the recruitment of mononuclear cells. The importance of MIP-1α in pathogenesis has been demonstrated in animal models of SLE, where blocking the chemokine was associated with amelioration of disease^35^. Mononuclear cells and their effector cytokines possibly act on the blood-brain barrier and the neuronal tissue leading to varied neurological manifestations, but the exact pathways need to be defined.

Autoantibodies are a hallmark of lupus, with several associations with clinical phenotypes. Our study showed 4 different clusters, while studies from various SLE cohorts have shown variable number of ranging from 2 to 5^25–27^. To make a simple tool for bedside use by the clinician, we studied the association between clinical phenotype, autoantibody profile, and cytokines. All 4 clusters had patients with nephritis, but cluster 3 with anti-Ro and nephritis appeared to be a rare observation. Although the number of patients in this cluster was small, it presents us with a clinical conundrum of an association between anti Ro and renal involvement. This association of anti-Ro/SSA autoantibody with lupus nephritis has been reported from Chinese and Korean cohorts showing Anti-Ro to be present in nearly two-thirds of both paediatric and adult lupus nephritis patients^36–38^. These observations taken together with the American data of Lupus nephritis showing Anti-Ro to be associated with ESRD, in a multivariate analysis, indicate a possible role of Anti-Ro antibodies besides the conventional autoantibodies like anti ds DNA, anti-nucleosome, anti-Ribosomal P and anti-C1q in Nephritis^39^.In addition, the low levels of housekeeping cytokines like Flt-3, GRO, IL-1RA, IL-8, IL-9, and IP-10 in the same cluster are possible indicators towards a novel group of patients. There are two reports presenting various autoantibody clusters and associated cytokines in SLE with contrasting results - the Columbian cohort of three clusters each with their autoantibodies correlating with active disease^28^ compared to the larger Swedish cohort having five autoantibody clusters but no difference with cytokine signature between them^29^. Our results are novel because of the assessment of clinical manifestations along with cytokine signatures and autoantibody clusters. There was a clear segregation for nephritis whereas no such pattern was observed for NPSLE. It could be due to a smaller number of patients with neurological manifestations like seizures and psychosis.

It is now realized that lupus is a heterogeneous disease with different pathways contributing to various clinical phenotypes^2^. The use of machine learning algorithms is providing newer insights into autoimmune disease pathogenesis and in the future will guide therapy. The major hindrance at present with the use of better technology is the lack of translation to bedside care especially for low to medium-income countries. The results of this pilot study provide hope that by using affordable laboratory tests one can predict organ involvement and severity of disease in SLE.

However, our study is not without limitations. The sample size was small, and patients were from a well-defined geographical area. The severe clinical manifestations were limited because of the small cohort. Another potential limiting factor could be our choice of prediction algorithm. Although PLSR is a well-established and widely used technique to infer predictive biomarker signatures, it is by no means the only choice. However, our decision to use PLSR was motivated by the fact that it is a simple and easily interpretable linear model. More complex nonlinear models may have had better predictive power, but because of our limited sample size, reliable sample splitting into training and validation sets would have been tricky, leading to overfitting. The results of our study need to be validated with a larger and more diverse patient population and the outcome could be the beginning of a new approach in understanding SLE.

## Methods

### Recruitment of patients

Sixty-seven (67) adult SLE patients attending the Department of Clinical Immunology and Rheumatology, SCB Medical College and Hospital, Cuttack, Odisha, India fulfilling SLICC criteria were recruited between January 2021 to January 2022 after taking informed consent. Baseline characteristics were recorded for all patients using a predesigned proforma. SLEDAI-2 K and SLICC ACR DI were used for the estimation of disease activity and damage at the point of entry. 11 age-matched healthy controls were recruited for comparison. The study was approved by the Institutional Ethics Committee of SCB Medical College and Hospital, Cuttack, and written informed consent was obtained from all participants. All the experimental procedures were carried out in accordance with institutional safety and regulatory guidelines.

The following patients were excluded from the study:

- Juvenile SLE.
- SLE patients with active infection.
- SLE patients with End-stage renal disease, Chronic liver disease, and Congestive cardiac failure.

### Measurement of ANA, anti-ds DNA, C3, C4 and ANA profile

ANA was assessed by Hep-2 method using commercially available kits (Euroimmun, Germany). Anti-dsDNA levels were measured using ELISA (Euroimmun, Germany). C3 and C4 levels were estimated using turbidimetric method (Spinreact, Germany) and line immunoassay was done for ANA profile (Euroimmun, Germany).

### Measurement of cytokines in plasma

Plasma was isolated from whole blood and stored at -80 °C until use. Cytokine measurements in plasma were performed by a commercially available human cytokine/chemokine multiplex kit (Millipore, USA). The following analytes were measured: sCD40L, EGF, Eotaxin/CCL11, FGF-2, Flt-3 ligand, Fractalkine, G-CSF, GM-CSF, GRO, IFN-α2, IFN-γ, IL-1α, IL-1β, IL-1RA, IL-2, IL-3, IL-4, IL-5, IL-6, IL-7, IL-8, IL-9, IL-10, IL-12 (p40), IL-12 (p70), IL-13, IL-15, IL-17 A, IP-10, MCP-1, MCP-3, MDC (CCL22), MIP-1α, MIP-1β, PDGF-AA, PDGF-AB/BB, RANTES, TGF-α, TNF-α, TNF-β, and VEGF. All procedures were carried out according to the manufacturer’s instructions. Readings were taken on a Bioplex 200 machine (BioRad Laboratories).

### Data preprocessing

We used background-subtracted fluorescence intensities of the cytokines for all our analyses. Initial inspection of the cytokines across all samples revealed that 7 samples out of the total of 67 had more than 10% of the measured cytokine values as missing. These samples were dropped from further analysis. Missing values in other groups were imputed by substituting with the column’s minimum value. A log(*n* + 1) transformation was applied to all the values to normalize the distributions.

### Partial least squares analysis (PLS)

PLS was performed either to discriminate patient categories (PLS-DA) based on combined cytokine response or to predict disease activity scores (PLS-R) from cytokine measurements. PLS-DA involves the calculation of latent variables (LVs) and loading vectors (coefficients) to create a model that best explains the relationship between X (cytokine measurements) and Y (patient categories or SLEDAI scores) while maximizing class separation. Both PLS-DA and PLSR were implemented via Python’s scikit-learn library (v 1·2·2).

### kModes clustering and multiple correspondence analysis (MCA)

kModes clustering was performed to cluster SLE patients according to their autoantibody profiles. kModes is a modification of the traditional k Means algorithm in that it uses the mode of the categorical attributes to define the cluster centers instead of means. Out of the 67 patients recruited, 35 patients had autoantibody profiles available, which were included in the analysis. For visualization of the clusters, we performed dimensionality reduction by multiple correspondence analysis (MCA), followed by projections of the patients on the first two dimensions. The samples were then coloured according to their kModes cluster. Package *kmodes* was used for kModes clustering and MCA was performed with the *Prince* package (https://maxhalford.github.io/prince/mca/). All analysis was done on Python 3·9·13.

### Statistical analysis

Univariate comparisons between groups were performed by Mann-Whitney U-test followed by Bonferroni’s correction for multiple comparisons. For statistical inference and subsequent plotting, we used the *statannotations* package for Python (https://github.com/trevismd/statannotations). All plots were created in Python using matplotlib (v3·7·1) and seaborn (v0·12·2) libraries.

## Electronic supplementary material

Below is the link to the electronic supplementary material.

Supplementary Material 1

## Data Availability

Some data and all scripts for conducting the analyses in this study can be shared upon reasonable request to the corresponding author.
