# Supplementary material for: Machine learning identifies cytokine signatures of disease severity and autoantibody profiles in systemic lupus erythematosus – a pilot study
Source: Sci Rep. 2024 Nov 20;14:28765. doi: 10.1038/s41598-024-79978-9 (PMC11579361; doi:10.1038/s41598-024-79978-9)
Supplement: Supplementary file 1 — Supplementary Material 1 [file 41598_2024_79978_MOESM1_ESM.pdf]

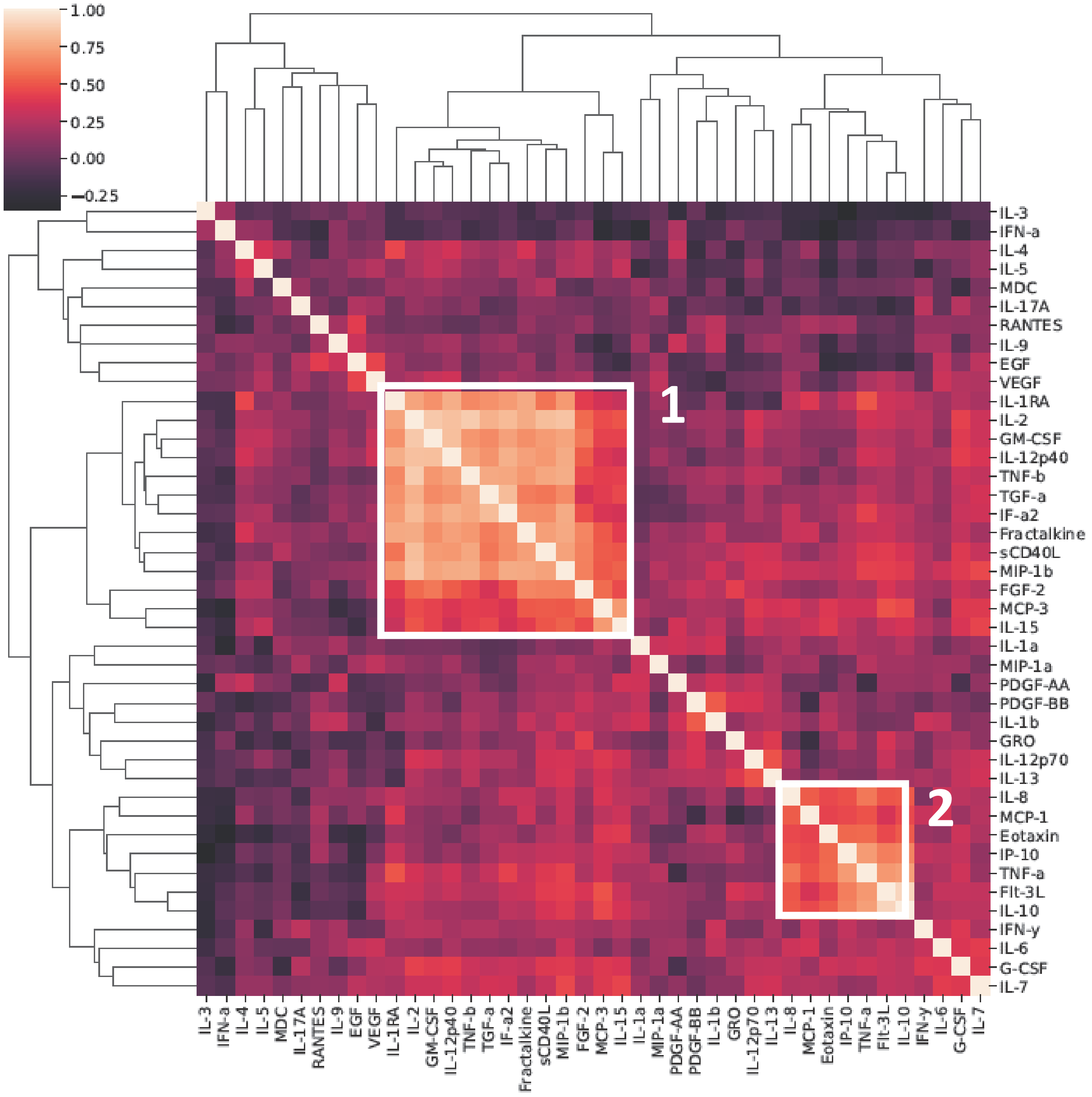

Supplementary Figure S1 Pairwise correlation matrix of measured cytokines in circulation. Colours in the heatmap depict Spearmann's correlation values. Two clusters of highly correlated cytokines can be seen.

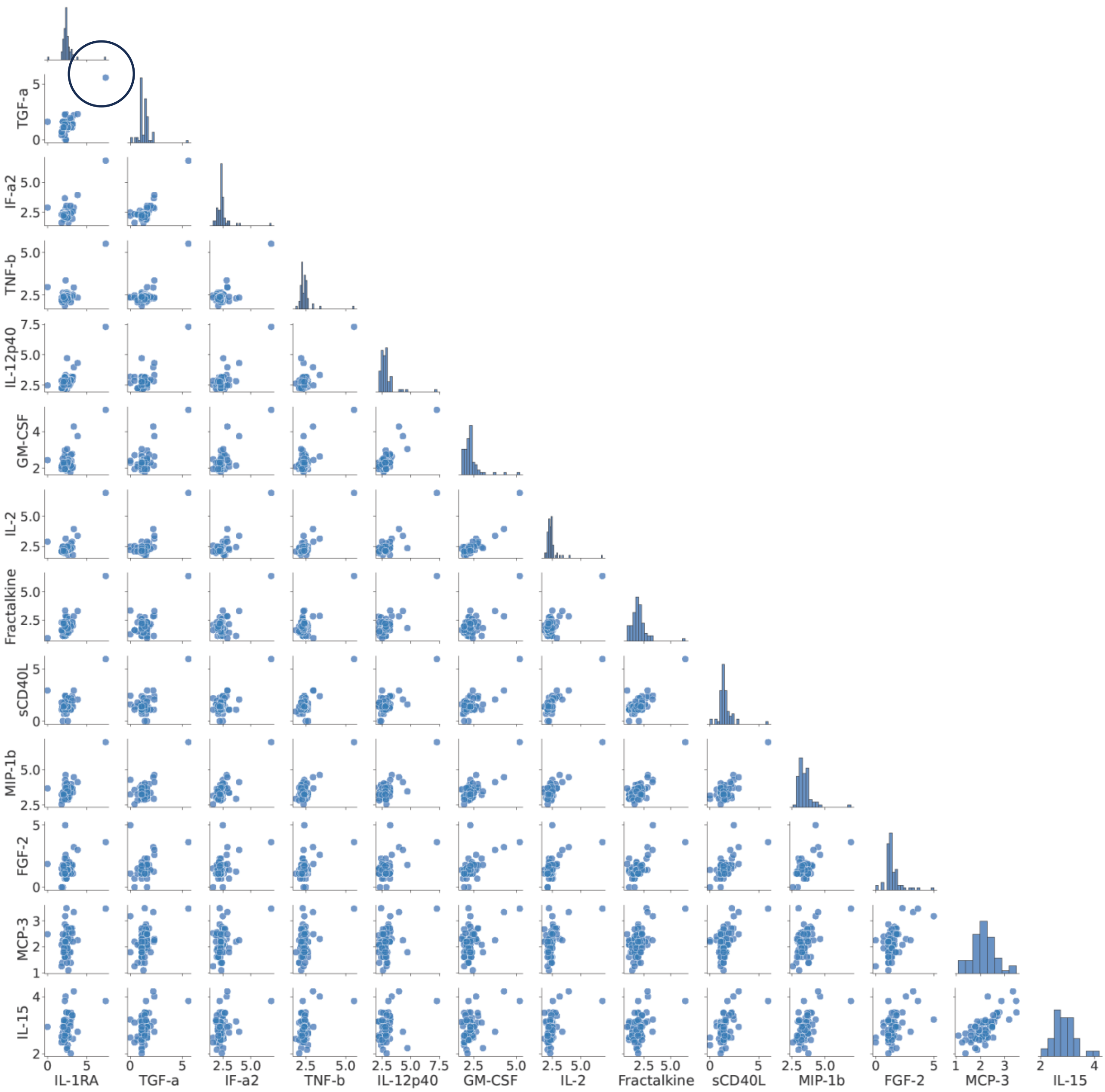

Supplementary Figure S2 Pairwise scatterplot of positively associated cytokines. Cytokines from cluster 1 identified in supplementary figure S1 was plotted on a 2X2 scatterplot matrix to identify potential outliers (circled).

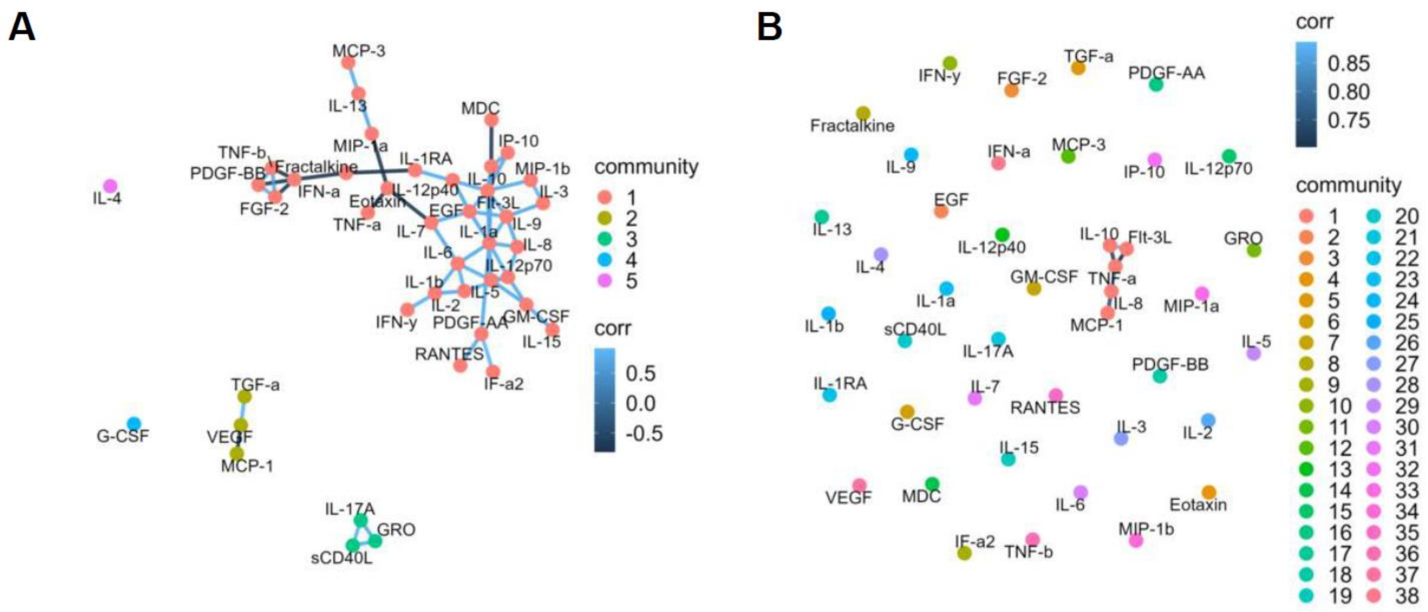

Supplementary Figure S3 SLE is associated with a global disruption of cytokine networks. (A) Correlations between circulating cytokines in healthy volunteers plotted as a network graph. **(B)** The same as in (A), but for patients with SLE. The nodes are the individual cytokines, coloured according to the cluster they fall in. The edges are spearman correlation values. The associated colour bar displays the strength of the correlation.

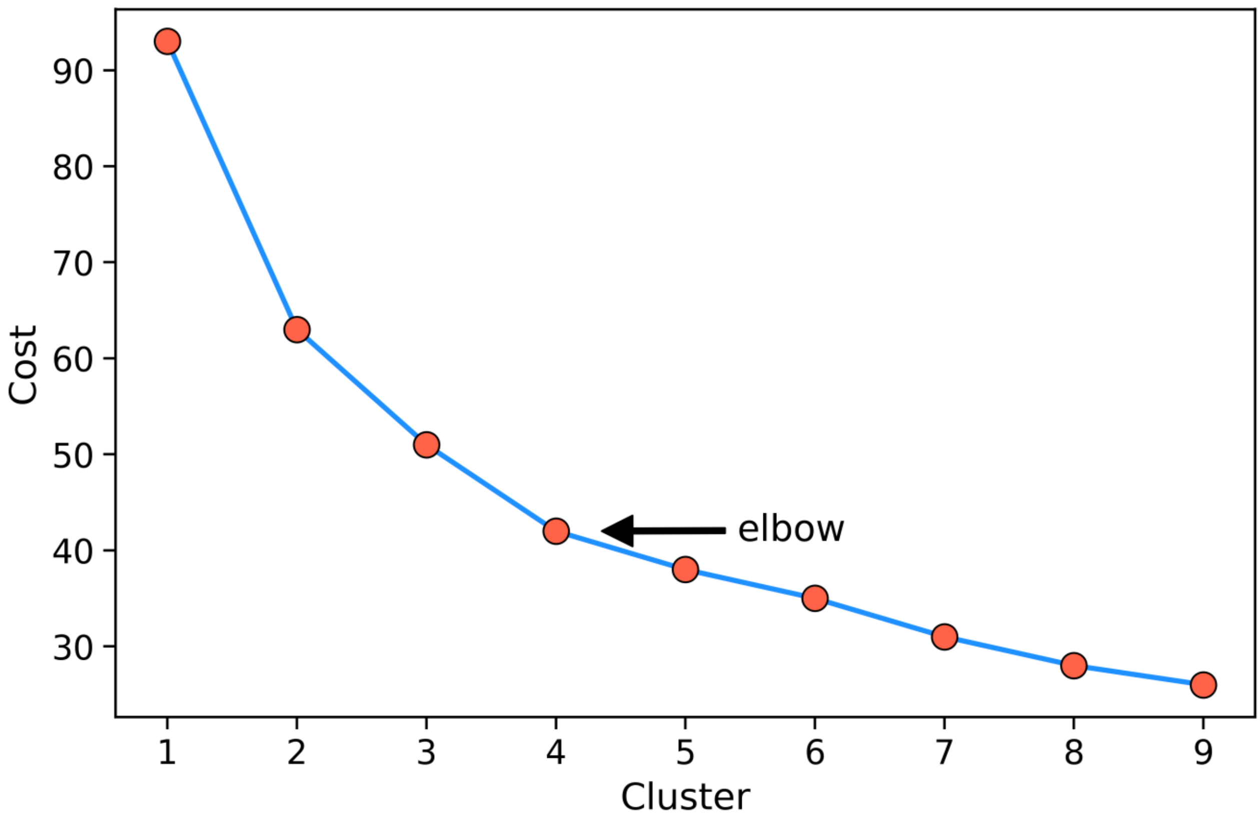

Supplementary Figure S4 Elbow plot to identify the optimum number of clusters for kModes algorithm.

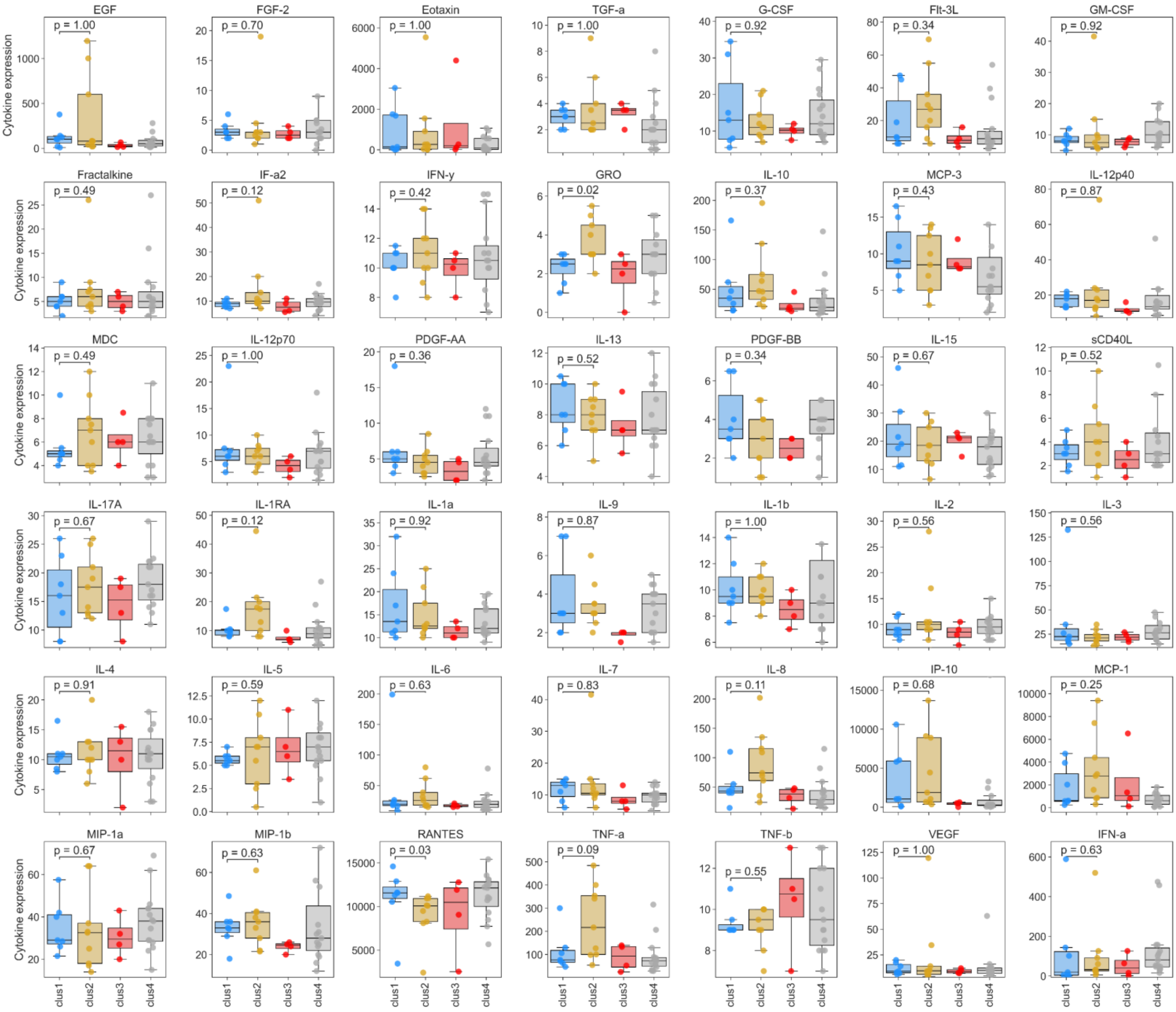

Supplementary Figure S5 Comparison of cytokine expression between patient clusters identified by autoantibodies.

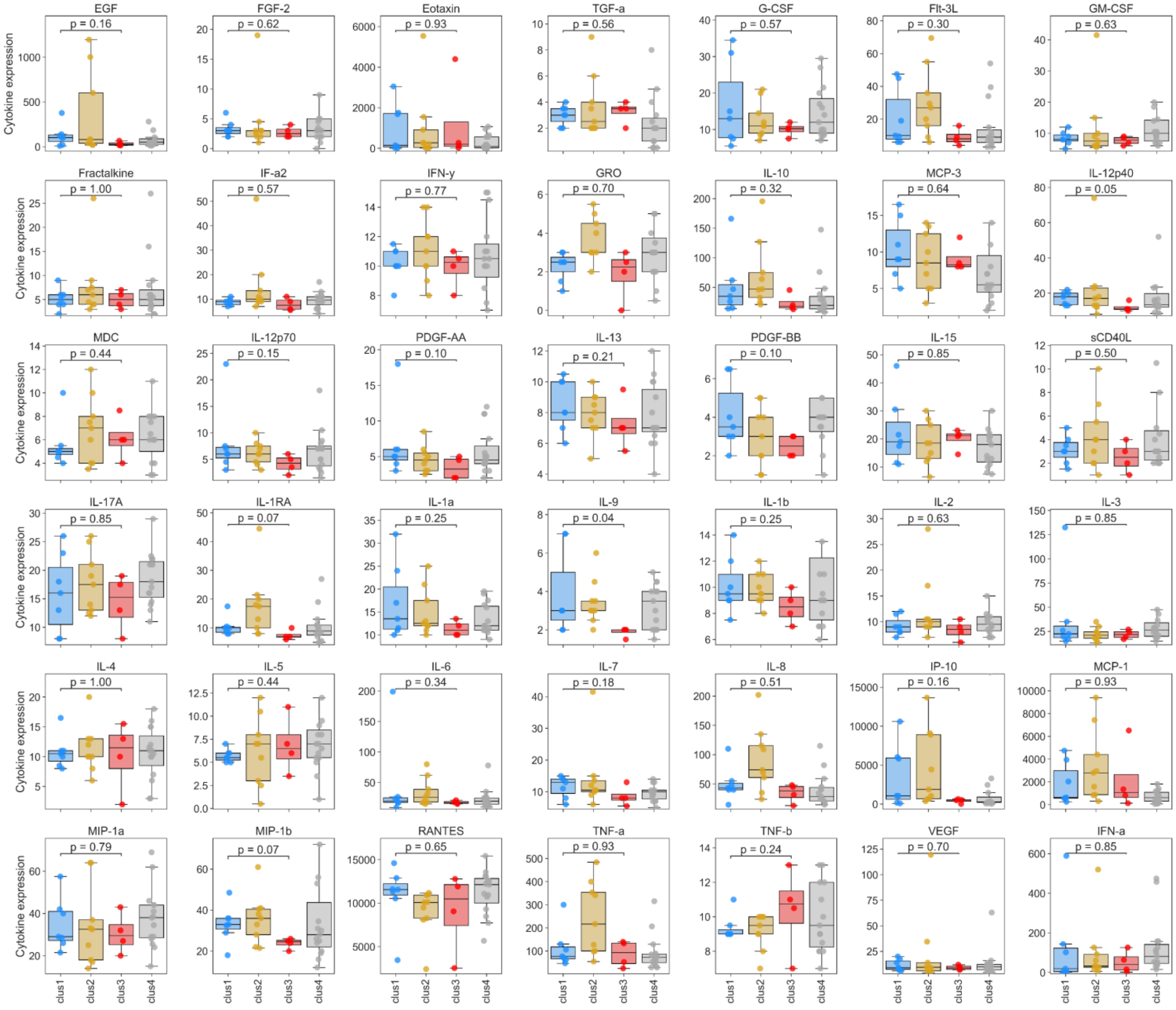

Supplementary Figure S6 Comparison of cytokine expression between patient clusters identified by autoantibodies.

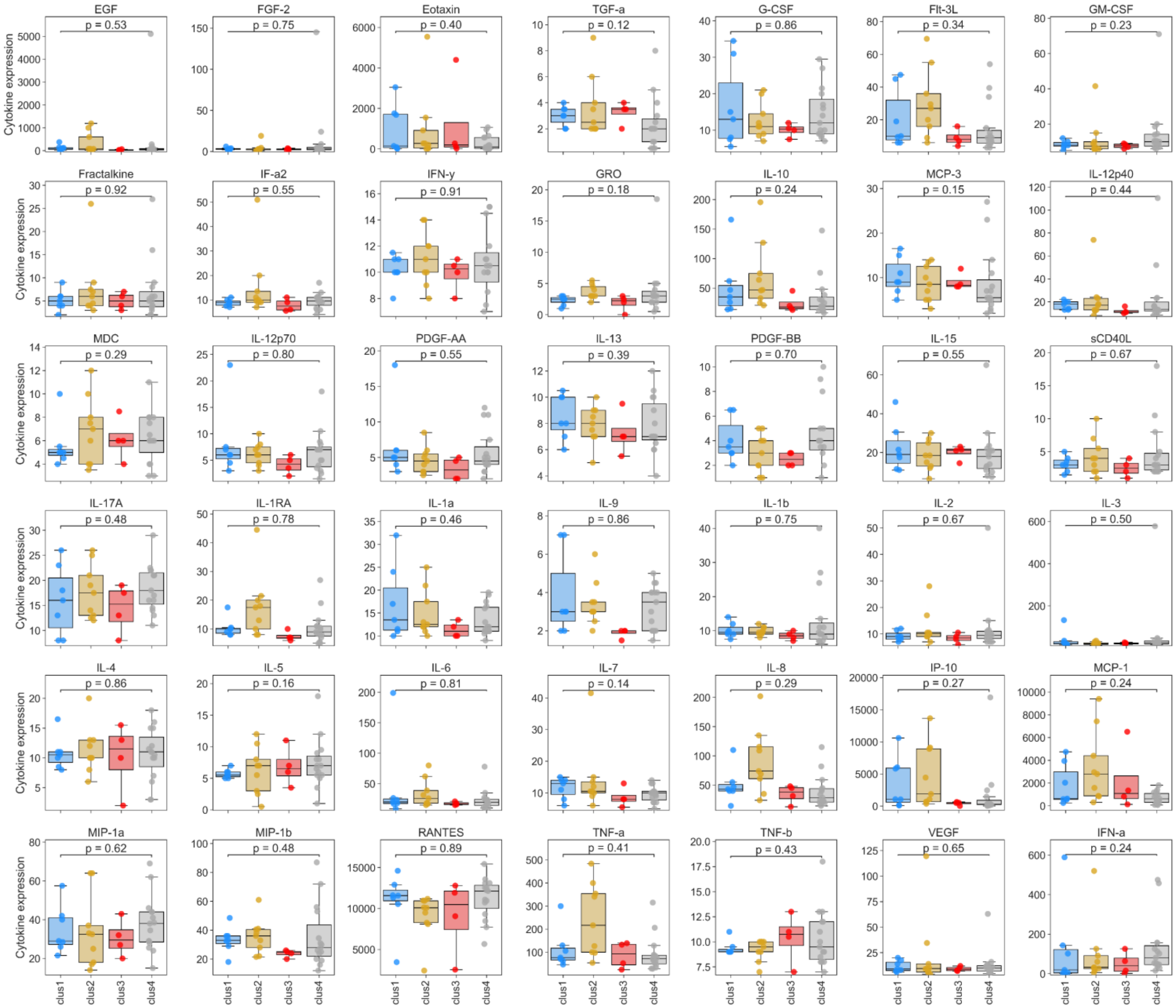

Supplementary Figure S7 Comparison of cytokine expression between patient clusters identified by autoantibodies.

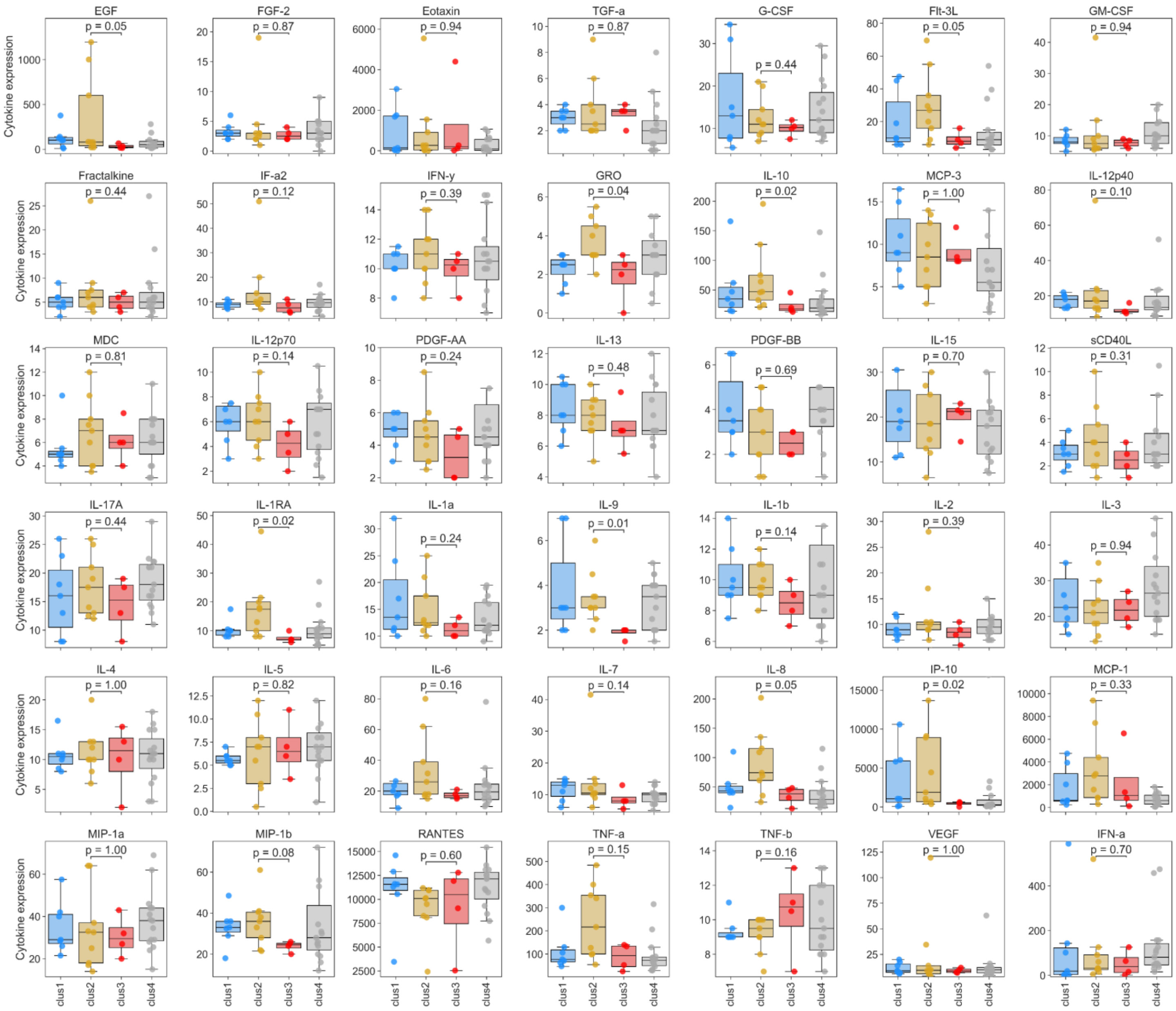

Supplementary Figure S8 Comparison of cytokine expression between patient clusters identified by autoantibodies.

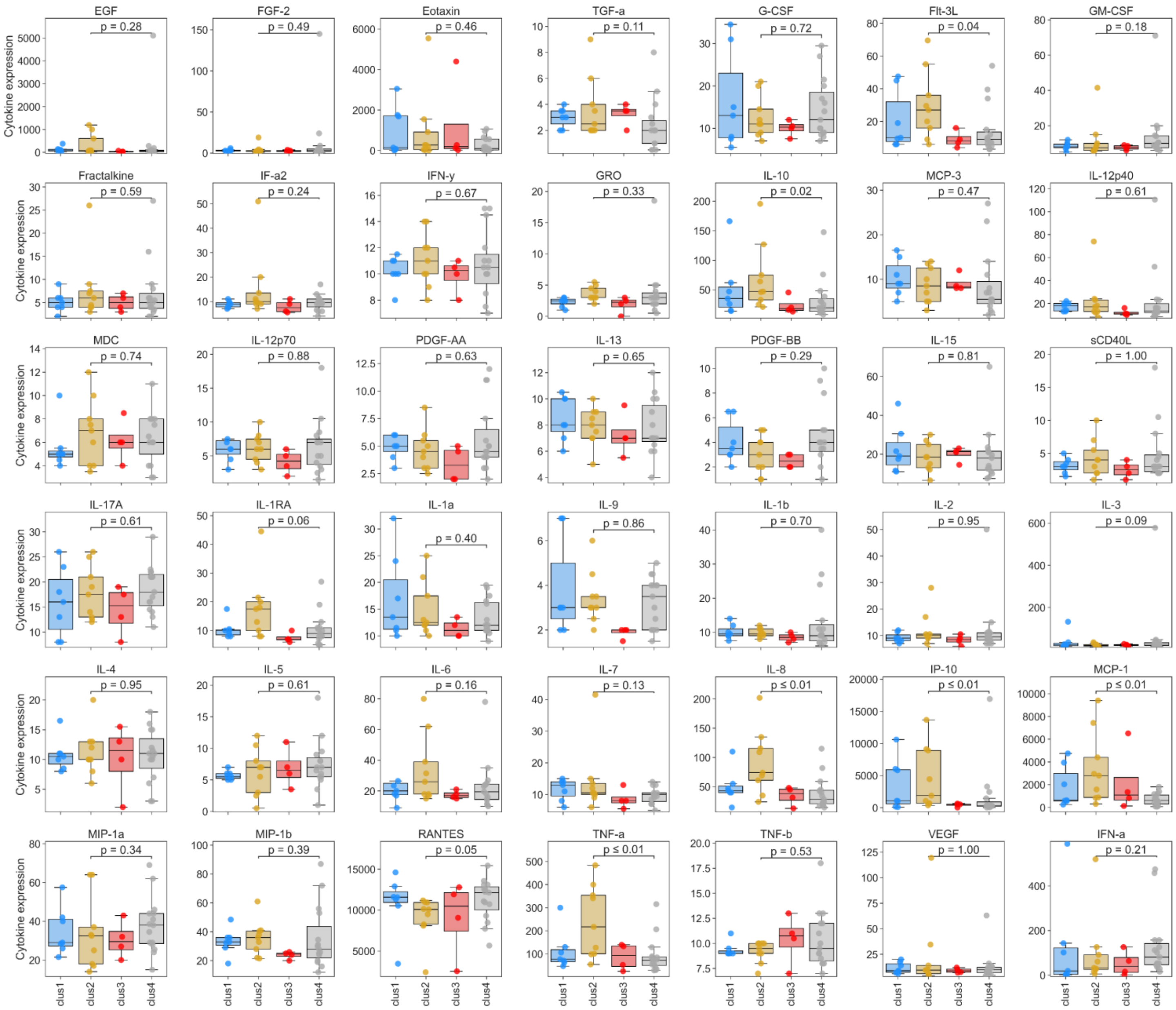

Supplementary Figure S9 Comparison of cytokine expression between patient clusters identified by autoantibodies.

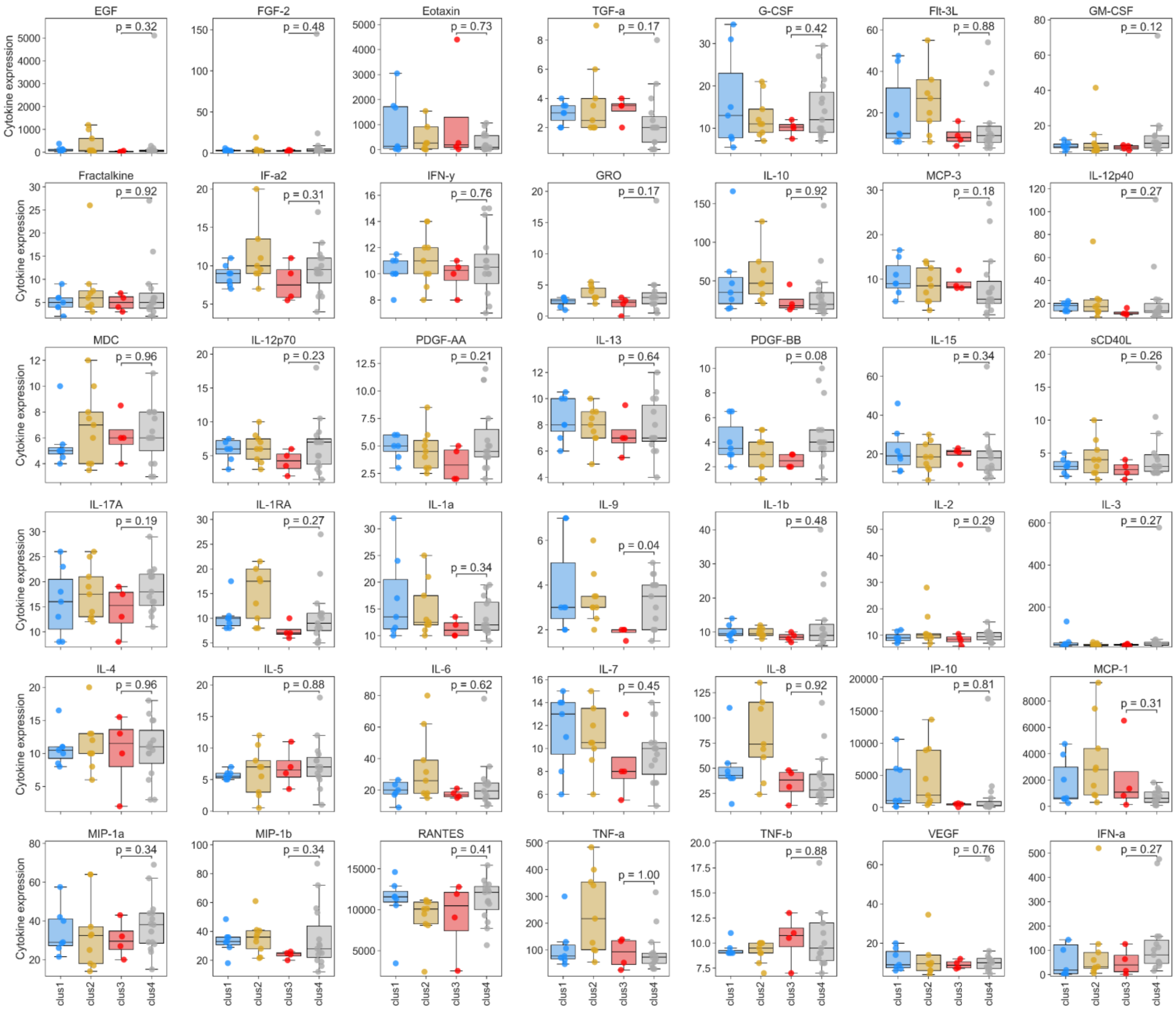

Supplementary Figure S10 Comparison of cytokine expression between patient clusters identified by autoantibodies.
